# Clinical Characteristics, Management, and Outcomes of Suspected Poststroke Acute Coronary Syndrome

**DOI:** 10.1155/2017/3762149

**Published:** 2017-10-09

**Authors:** Sylvia Marie Biso, Marvin Lu, Toni Anne De Venecia, Supakanya Wongrakpanich, Mary Rodriguez-Ziccardi, Sujani Yadlapati, Marina Kishlyansky, Harish Seetha Rammohan, Vincent M. Figueredo

**Affiliations:** ^1^Department of Medicine, Einstein Medical Center, 5401 Old York Road, Suite 363, Philadelphia, PA 19141, USA; ^2^Bassett Medical Center, Bassett Healthcare Network, Cooperstown, NY, USA; ^3^CUMC, College of Physicians & Surgeons, Columbia University, New York, NY, USA; ^4^Einstein Institute for Heart and Vascular Health, Einstein Medical Center, Philadelphia, PA, USA; ^5^Sidney Kimmel Medical College, Thomas Jefferson University, Philadelphia, PA, USA

## Abstract

**Background:**

Acute coronary syndrome (ACS) can complicate acute ischemic stroke, causing significant morbidity and mortality. To date, literatures that describe poststroke acute coronary syndrome and its morbidity and mortality burden are lacking.

**Methods:**

This is a single center, retrospective study where clinical characteristics, cardiac evaluation, and management of patients with suspected poststroke ACS were compared and analyzed for their association with inpatient mortality and 1-year all-cause mortality.

**Results:**

Of the 82 patients, 32% had chest pain and 88% had ischemic ECG changes; mean peak troponin level was 18, and mean ejection fraction was 40%. The medical management group had older individuals (73 versus 67 years, *p* < 0.05), lower mean peak troponin levels (12 versus 49, *p* < 0.05), and lower mean length of stay (12 versus 25 days, *p* < 0.05) compared to those who underwent stent or CABG. Troponin levels were significantly associated with 1-year all-cause mortality.

**Conclusion:**

Age and troponin level appear to play a role in the current clinical decision making for patient with suspected poststroke ACS. Troponin level appears to significantly correlate with 1-year all-cause mortality. In the management of poststroke acute coronary syndrome, optimal medical therapy had similar inpatient and all-cause mortality compared to PCI and/or CABG.

## 1. Introduction

Acute coronary syndrome can complicate acute ischemic stroke. In the Randomized Trial of Tirilazad Mesylate in Patients with Acute Stroke (RANTTAS trial), cardiac ischemia was found to occur in 6% of patients, with 1% being life-threatening [1]. A study by Chin found that acute coronary syndrome occurred in 12.7% of patients within 3 days of an acute ischemic stroke [2]. Cardiac abnormalities also were found to be the most common cause of death after stroke. Six percent of unexpected deaths in these cases occurred in the first month [3]. Even 3.5 years after a stroke, patients were still found to have an annual 2% risk of myocardial infarction [4].

Neurologic events are known to cause myocardial injury and dysfunction. Cardiac injury after an acute stroke has been shown to occur even in the absence of underlying coronary artery disease [5]. There is a strong association between cerebrovascular disease and coronary artery disease [4]: Both stroke and coronary artery disease have the same risk factors [6] and there is high prevalence of cardiac disease among stroke patients [7]. However, the literature is scant, not only in describing patients with ischemic brain infarct and acute coronary syndrome, but also in regard to their evaluation and medical management. Current practice for myocardial infarction after a stroke is a full cardiac work-up and coronary angiography is needed to rule out significant coronary artery disease [8, 9].

Given the lack of guidelines and literature for poststroke ACS patients, the objectives of this study were to describe the demographics and comorbidities of patients with poststroke acute coronary syndrome, including their presenting symptom, peak troponin level, presence of ischemic electrocardiogram (ECG) changes, 2D-echocardiograms, and cardiac catheterization results. We compared the clinical characteristics and cardiac evaluation of patients who were medically managed and those who underwent any invasive intervention with percutaneous coronary intervention (PCI) or coronary artery bypass graft surgery (CABG). We sought to determine if there is a difference in inpatient mortality and 1-year all-cause mortality among these patients.

## 2. Methodology

This is a retrospective cohort study on adult patients who were admitted for acute ischemic stroke at Albert Einstein Medical Center from January 2003 to December 31, 2013, and developed acute coronary syndrome within 72 hours after ischemic stroke. Acute coronary syndrome referred to patients who fulfilled the third universal definition criteria of elevated cardiac biomarkers of at least one value above the 99th percentile upper reference limit and one of the following: symptoms of ischemia, development of pathologic Q waves in the electrocardiogram, new or presumed new significant ST-segment-T-wave (ST-T) changes or new left bundle branch block (LBBB), and imaging evidence of new loss of viable myocardium or a new regional wall motion abnormality. We excluded the patients with age < 18 years, patients with intracerebral hemorrhage, subarachnoid hemorrhage, hemorrhagic contusions, epidural hemorrhage, subdural hemorrhage, and other brain lesions, and patients with end stage renal disease on hemodialysis.

Baseline demographic data (age, gender, race, and body mass index) and comorbidities such as preexisting coronary artery disease, hypertension, hyperlipidemia, diabetes mellitus, chronic kidney disease, congestive heart failure (systolic and/or diastolic heart failure), coronary artery disease equivalents (i.e., peripheral artery disease and carotid artery disease), and smoking history were collected. Results of cardiac evaluation such as peak troponin level, ECG changes, 2D-transthoracic echocardiograms, and cardiac catheterization were also obtained. The primary outcomes of the study were inpatient mortality and 1-year all-cause mortality from the time of the acute ischemic stroke. The mortality information was acquired using social security database index (SSDI).

For data analysis, categorical data were presented as numbers and percentages and continuous data as mean ± standard deviation. The demographic characteristics, comorbidities, and cardiac evaluation results of those who were medically managed versus those who underwent cardiac catheterization and subsequent stent placement or CABG were compared using Student's *t*-test to test for differences between independent continuous variables and the chi-square test to test for differences between categorical variables. For continuous data that are not normally distributed, a nonparametric test will be used. For example, a rank-sum test (Kruskal-Wallis test) will be used instead of *t*-test. In a 2 × 2 table, if one of the cells contains an expected value of less than 5, Fisher exact test will be used instead of chi-square test. To measure the association between an outcome variable (inpatient mortality and 1-year all-cause mortality) and selected exposure variable, such as peak troponin level, ischemic changes in ECG, and intervention, the relative risk will be computed. The chi-square or Fisher exact test will be used to determine the significance of the association.

## 3. Results

Among adult patients admitted to Albert Einstein Medical Center from 2003 to 2013, there were 82 patients who had an acute coronary syndrome after an acute ischemic stroke. The mean age was 72 years; 52% were female. Sixty-two percent of the patients in the study were African American, 30% were Caucasian, 6% were Hispanic, and 2% were Asian (Table 1).

Most of the patients in the study were found to have hypertension (96%). The next most common comorbidities were diabetes mellitus (56%), hyperlipidemia (45%), known coronary artery disease (44%), and congestive heart failure (46%). One-third of the population had a body mass index above 30 or chronic kidney disease. Around 20% were smokers or had coronary artery disease equivalents (Table 1).

Surprisingly, among patients who had acute coronary syndrome after an acute ischemic stroke, only 32% complained of chest pain. Other criteria for acute coronary syndrome occurred more frequently. Ischemic ECG changes (i.e., new T-wave inversions, new Q waves, new LBBB, ST-segment elevations, and ST-segment depressions) were found 88% of the time. The most common ischemic ECG change was new T-wave inversion (78%). Mean peak troponin level was 18. Wall motion abnormalities on 2D-echocardiograms occurred in 43% of the patients with 88% in coronary artery territories corresponding to the ischemic ECG changes. The mean ejection fraction of the patients in the study was 40% (Table 2).

Although the patients had acute coronary syndromes, 67% did not undergo cardiac catheterization and were managed conservatively. Of the 33% who underwent cardiac catheterization, 15% had no significant coronary artery disease, 26% had 1-vessel disease, 15% had 2-vessel disease, and 44% had 3-vessel disease. Overall, out of the 82 patients, only 16% underwent placement of stents or CABG. The remaining 84% were managed medically. Medical management was in accordance with the guideline-directed medical therapy for acute coronary syndromes and congestive heart failure by the American College of Cardiology/American Heart Association guidelines that include aspirin, clopidogrel, and beta-blockers. For those with reduced ejection fraction, ACE inhibitors, beta-blockers, hydralazine/nitrates, and so on (depending on patient's clinical status) were given. The mean length of stay was 14 days (Table 2).

The medical management group was significantly older (73 versus 67 years, *p* < 0.05) and had a significantly lower length of stay (12 versus 25 days, *p* < 0.05) (Table 3). In terms of comorbidities, the two groups were the same (Table 3). As for markers of cardiac injury, the mean peak troponin level for the medical management group was 12 compared to the intervention group, which was 49. There was no significant difference between the two when it comes to presence of ischemic ECG changes and 2D-echo results (i.e., mean ejection fraction and presence of wall motion abnormality) (Table 4).

There was a trend towards inpatient mortality among patients who were managed medically compared to the intervention group, although this was not significantly different (32% versus 23%, *p* > 0.05). The 1-year all-cause mortality, however, was similar (52% versus 54%, *p* > 0.05) (Tables 5(a) and 5(b)).

## 4. Discussion

The main findings of this study show that age and troponin level appear to play a role in the current clinical decision making for patients with suspected poststroke ACS. Troponin level appears to significantly correlate with 1-year all-cause mortality. In the management of poststroke acute coronary syndrome, optimal medical therapy had similar inpatient and all-cause mortality compared to PCI and/or CABG. Although there was a trend towards inpatient mortality among patients who were managed medically compared to the intervention group, it was not found to be statistically significant (*p* > 0.05) (Tables 5(a) and 5(b)). The 1-year all-cause mortality rates were also found to be similar between the two groups, with 52% for the medical management groups and 54% for the intervention group.

Most patients who had acute coronary syndrome after an acute ischemic stroke did not have any symptoms that pertain to the ACS. Only around 30% had chest pain. This may be explained by language or cognitive impairments that occur in the setting of acute ischemic stroke. Alternatively, it is possible that many of the cases presented as a silent myocardial infarction. The patients' troponin levels, however, reflect a significant amount of cardiac injury, being remarkably elevated to a mean of 18. This emphasizes the importance of cardiac evaluation in acute ischemic stroke patients, even without obvious typical symptoms.

In a study by Chalela et al., troponin elevation was found to occur in 6% of patients with acute ischemic stroke. In another study, 53.4% of the patients admitted for acute ischemic stroke have elevated troponin, although only 6.6% met the criteria for acute coronary syndrome [10]. Interestingly, the study also showed that elevated troponin levels correlated with older age, history of coronary artery disease, congestive heart failure, diabetes mellitus, and renal disease [11]. An elevated troponin level was also associated with severity of stroke [10], poor outcomes, and death [12]. Troponin levels were associated with predicting a modified Rankin score, a measure to determine the degree of disability in doing activities of daily living, of 5 (bedridden) or 6 (death) [12]. Furthermore, a study by Scheitz et al. showed that an elevated troponin level was an independent predictor of poor outcome and in-hospital mortality [13]. In our study, the troponin levels revealed being significantly related to 1-year all-cause mortality, with 15 out of the 82 patients dying within 1 year of the ischemic stroke (Tables 5(a) and 5(b)). In contrast to Scheitz's study, our data did not show that troponin level is associated with inpatient mortality. However, because results across multiple studies have been different, it remains to be determined if troponin level correlates only with the severity of stroke or whether it is an independent predictor of death [14].

A clinical review by Davis et al. showed that the most frequent ECG abnormalities in acute stroke patients were prolonged QT interval and ST-T changes [15]. In a study by Dogan et al., on the other hand, 65% of patients had ischemia-like ECG changes with the most common one being T-wave inversion [16]. In our study, presence of ischemic ECG changes was found in 88% of the patients and similarly to Dogan's study the most common abnormality was T-wave inversion (89%). Although Dogan's study showed that ST-segment changes were an independent predictor of mortality, a large scale study by Goldstein showed that ischemic ECG changes were not associated with mortality in stroke patients [17]. Our study was consistent with Goldstein's results showing that ischemic ECG changes did not correlate with inpatient mortality or 1-year all-cause mortality (Tables 5(a) and 5(b)).

Our study revealed that patients who had acute coronary syndrome in the setting of acute ischemic stroke had low ejection fraction and wall motion abnormalities. The mean EF of our patients is 40, which is close to the results of a study by Bulsara et al., where the mean EF of the patients was 33%. In Bulsara's study, the findings in the ECGs did not match the wall motion abnormalities in the 2D-echocardiograms [18]. Our study, however, showed that wall motion abnormalities correlated with the ischemic changes found in the ECG (Table 2). In a study done with subarachnoid hemorrhage patients, wall hypokinesis was present in the first 2 days and there was partial or complete resolution of the wall motion abnormalities during the hospitalization [19]. Due to the retrospective nature of our study, we were not able to follow the 2D-echocardiograms of our patients during their hospital stay.

In our study, 67% of patients with acute coronary syndrome and acute ischemic stroke did not undergo cardiac catheterization. There are several factors that may have influenced this management decision. One is the size of the stroke. Patients who are deemed at risk of hemorrhagic conversion do not undergo cardiac catheterization during the same admission. The procedure may then be scheduled 3–6 months after the stroke. It is also important to note that 29% of the patients in the study had chronic kidney disease. In elderly patients, it is not uncommon to encounter patients and family members refusing cardiac catheterization because of higher risk of requiring hemodialysis.

Our study also found that older age is associated with greater likelihood of conservative management (Table 3). This may be because the risks of cardiac catheterization sometimes outweigh the benefits in frail, elderly patients. The level of troponin also plays a role in deciding the management. The higher it is, the more likely the patient will undergo cardiac catheterization as this may imply larger cardiac damage (Table 4). Apart from the age and troponin level, the two groups (patients who were managed conservatively and those who underwent cardiac catheterization) did not have any significant difference in terms of comorbidities (Table 3). Since the cooccurrence of acute coronary syndrome and acute ischemic stroke is not common, this study is limited by its sample size. There is a trend seen in medical management group and inpatient mortality; however, the limited sample size may account for why it is not statistically significant.

In conclusion, when clinical characteristics and cardiac work-up of the medical management group and the intervention group were compared, the only significant difference was that medical management group had older individuals (73 versus 67 years), lower mean peak troponin levels (12 versus 49), and lower mean length of stay (12 versus 25 days). Troponin levels revealed being significantly related to 1-year all-cause mortality, but not to inpatient mortality. Optimal medical therapy had similar inpatient and all-cause mortality compared to PCI and/or CABG.

## 5. Limitations of the Study

Given the retrospective nature of our study, variables like severity of stroke, MRI findings for the location of the stroke, patient frailty, and other related variables were missing in our patient records and were not included in the study. Sixty-seven percent of the patients in the study also did not undergo cardiac catheterization. Finally, it is possible that the elevated troponin levels seen in our patient group were secondary to catecholamine surge caused by the stroke.

## Figures and Tables

**Table 1:** Demographic data of patients with poststroke acute coronary syndrome.

	Total (%)
*N* (%)	82
Mean age (years)	72 ± 12.5
Gender	
Female	43 (52%)
Male	39 (48%)
Race	
Caucasian	24 (30%)
African American	51 (62%)
Hispanic	5 (6%)
Asian	2 (2%)
Comorbidities	
Coronary artery disease	36 (44%)
Body mass index > 30	22 (33%)
Hypertension	79 (96%)
Hyperlipidemia	37 (45%)
Diabetes mellitus	46 (56%)
Chronic kidney disease	24 (29%)
Congestive heart failure	38 (46%)
Coronary artery disease equivalents	15 (18%)
Smoker	20 (24%)

**Table 2:** Clinical data and management of patients with poststroke acute coronary syndrome.

	Total (%)
Chest pain	26 (32%)
Mean peak troponin level	18 ± 33
ECG: presence of ischemic ECG changes	72 (88%)
Echo	
Mean EF%	40 ± 20
Presence of wall motion abnormalities	35 (43%)
Corresponding to ischemic ECG changes	72 (88%)
Not corresponding to ischemic ECG changes	10 (12%)
Cardiac catheterization	
Did not undergo cardiac catheterization	55 (67%)
Underwent cardiac catheterization	27 (33%)
Cardiac catheterization results:	
No significant coronary artery disease	4 (15%)
1-vessel disease	7 (26%)
2-vessel disease	4 (15%)
3-vessel disease	12 (44%)
Management	
Medical management	69 (84%)
Stent or CABG	13 (16%)
Mean length of stay (days)	14 ± 13

**Table 3:** Patient characteristics of those who underwent medical management versus intervention (stent or CABG).

	Medical management	Stent or CABG	*p* value
*N* (%)	69 (84%)	13 (16%)	
Mean age (years)	73 ± 13	67 ± 7	*p* = 0.02
Gender			
Female	38 (88%)	5 (12%)	*p* = 0.21
Male	31 (80%)	8 (20%)	
Race			*p* = 0.6
Caucasian	20 (83%)	4 (17%)	
African American	42 (82%)	9 (18%)	
Hispanic	5 (100%)	0 (0%)	
Asian	2 (100%)	0 (0%)	
Mean length of stay (days)	12 ± 10	25 ± 21	*p* = 0.005
Comorbidities			
Coronary artery disease	29 (80%)	7 (20%)	*p* = 0.3
Body mass index > 30	17 (77%)	5 (23%)	*p* = 0.44
Hypertension	66 (83%)	13 (17%)	*p* = 0.5
Hyperlipidemia	32 (86%)	5 (14%)	*p* = 0.41
Diabetes mellitus	39 (85%)	7 (15%)	*p* = 0.5
Chronic kidney disease	20 (83%)	4 (17%)	*p* = 0.5
Congestive heart failure	30 (79%)	8 (21%)	*p* = 0.18
Coronary artery disease equivalents	12 (80%)	3 (20%)	*p* = 0.8
Smoker	15 (75%)	5 (25%)	*p* = 0.17

**Table 4:** Cardiac evaluation results of patients who underwent medical management versus intervention (stent or CABG).

	Medical management	Stent + CABG	Stat test	*p* value
Mean peak troponin	12 ± 17	49 ± 64	Kruskal-Wallis	0.005
Ischemic ECG changes				
With	62	10	Fisher exact	0.19
Without	7	3		
2D-echocardiogram				
Mean ejection	40 ± 18	38 ± 17	*t*-test	0.64
Wall motion abnormality				
Present	39	8	Chi-square	0.11
Absent	30	5		

**(a):** 

	Inpatient mortality		Inpatient mortality		RR	95% CI	Stat test	*p* value
	Number	%	Yes	No				
Mean peak troponin								
High	8	32	8	13	1.36	0.69–2.67	Chi-square	0.37
Low	17	68	17	44				
Ischemic ECG changes								
Present	8	32	8	13	1.3669	0.69–2.69	Fisher exact	0.26
Absent	17	68	17	44				
Management								
Stent/CABG	3	12	8	13	1.36	0.69–2.67	Chi-square	0.37
Medical	22	88	17	44				

**(b):** 

	1-year all-cause mortality		1-year all-cause mortality		RR	95% CI	Stat test	*p* value
	Number	%	Yes	No				
Mean peak troponin								
High	15	34.88	15	6	1.55	1.06–2.28	Chi-square	0.04
Low	28	65.12	28	33				
Ischemic ECG changes								
Present	38	88.37	38	34	1.05	0.54–2.03	Fisher exact	0.56
Absent	5	11.63	5	5				
Management								
Stent/CABG	7	16.28	7	6	1.03	0.59–1.7	Chi-square	0.57
Medical	36	83.72	36	33				
